# Spontaneous Rectal Perforation in a Patient with SARS–CoV-2 Infection

**DOI:** 10.3390/jpm10040157

**Published:** 2020-10-08

**Authors:** Mauro Giuffrè, Alessandro Marco Bozzato, Stefano Di Bella, Alessandro Agostino Occhipinti, Paola Martingano, Marco Francesco Maria Cavallaro, Roberto Luzzati, Fabio Monica, Maria Assunta Cova, Lory Saveria Crocè

**Affiliations:** 1 Department of Medical, Surgical and Health Sciences, University of Trieste, 34151 Trieste, Italy; alessandroj.bozzato@gmail.com (A.M.B.); stefano932@gmail.com (S.D.B.); roberto.luzzati@asuits.sanita.fvg.it (R.L.); m.cova@fmc.units.it (M.A.C.); lcroce@units.it (L.S.C.); 2 Italian Liver Foundation, 34149 Basovizza Trieste, Italy; 3 Emergency Department, Trieste University Hospital, 34139 Trieste, Italy; alexander82@tiscali.it; 4 Department of Radiology, Trieste University Hospital, 34139 Trieste, Italy; pmartingano@sirm.org (P.M.); marcofrancescomaria.cavallaro@asugi.sanita.fvg.it (M.F.M.C.); 5 Department of Gastroenterology, Trieste University Hospital, 34139 Trieste, Italy; fabio.monica@asuits.sanita.fvg.it

**Keywords:** COVID-19, SARS–CoV-2, fecal calprotectin, bowel perforation, radiology COVID-19

## Abstract

Coronavirus disease 2019 (COVID-19) is mostly perceived as a respiratory disease. However, there is increasing evidence of patients showing gastrointestinal symptoms, with increasing rates of presentation according to the severity of the disease. In a few cases, the abdominal involvement of COVID-19 resulted in spontaneous bowel perforation. Here, we present in detail the first case of rectal perforation in a patient with COVID-19.

## 1. Introduction

At the time of writing the present report, the leading cause of SARS–CoV-2-related mortality is respiratory failure, and coronavirus disease 2019 (COVID-19) is mostly perceived as a respiratory disease [1,2,3]. However, COVID-19 has significant extrapulmonary complications. In particular, gastrointestinal symptoms appear to affect 28% of patients with COVID-19 [4], with increasing rates of presentation according to the severity of the disease [5]. Additional findings include the detection of fecal viral RNA in around 50% of patients [6,7,8], the measurement of increased fecal calprotectin (FC) in patients, with or without gastrointestinal symptoms [9,10], and the detection of bowel wall abnormalities in 31% of computed tomography (CT) performed on patients with COVID-19 [11]. In a few cases, the abdominal involvement of COVID-19 resulted in spontaneous bowel perforation [9,11,12,13], and, to the best of our knowledge, no rectal perforation has ever been described before. Here, we present, in detail, the first case of rectal perforation in a patient with COVID-19.

## 2. Case Report

An 87-year-old woman living in a nursing home was admitted to the emergency department with abdominal pain, cough, and high-grade fever, which had started four days prior to hospital admission.

Her past medical history included polymyalgia rheumatica, giant cell arteritis, and slight cognitive impairment. She had no previous history of abdominal surgery nor gastrointestinal symptoms. The patient did not report any contact with people at high-risk for SARS–CoV-2 infection. However, real-time PCR of nasopharyngeal swab tested positive for SARS–CoV-2.

On admission, her blood pressure was 100/60 mmHg, her pulse was 100 bpm rhythmic, and SatO_2_ was 95% in ambient air and 97% in O_2_ at 40%.

Physical examination of the chest revealed a reduction of murmur to the pulmonary bases, bilaterally. In addition, the whole abdomen appeared distended, diffusely tender, and positive for Blumberg’s sign. Bowel sounds were not audible. Moreover, a rectal examination revealed rectorrhagia.

Laboratory tests were as follows: white blood cell count 5990 cells/mm^3^, hemoglobin 15 g/dL, platelet count 233.000 10^9^/L, C-reactive protein 290 mg/dL, INR 1.22, aPTT 29.2 s, fibrinogen 790 mg/dL, D-Dimer 2.1 mg/LFEU, fecal calprotectin 290 mg/kg. Renal, liver, and pancreatic function tests were within the range of normality.

**Figure 1:**
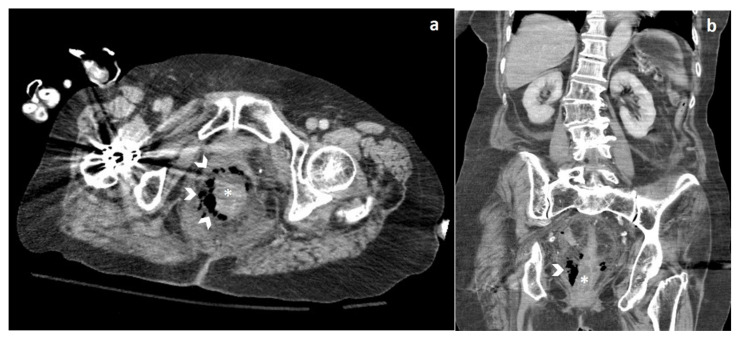
Axial (**a**) and coronal (**b**) computed tomography (CT) images show wall thickening (asterisk) of the lower-third of the rectum, surrounded by extraluminal free air (arrowheads).

**Figure 2:**
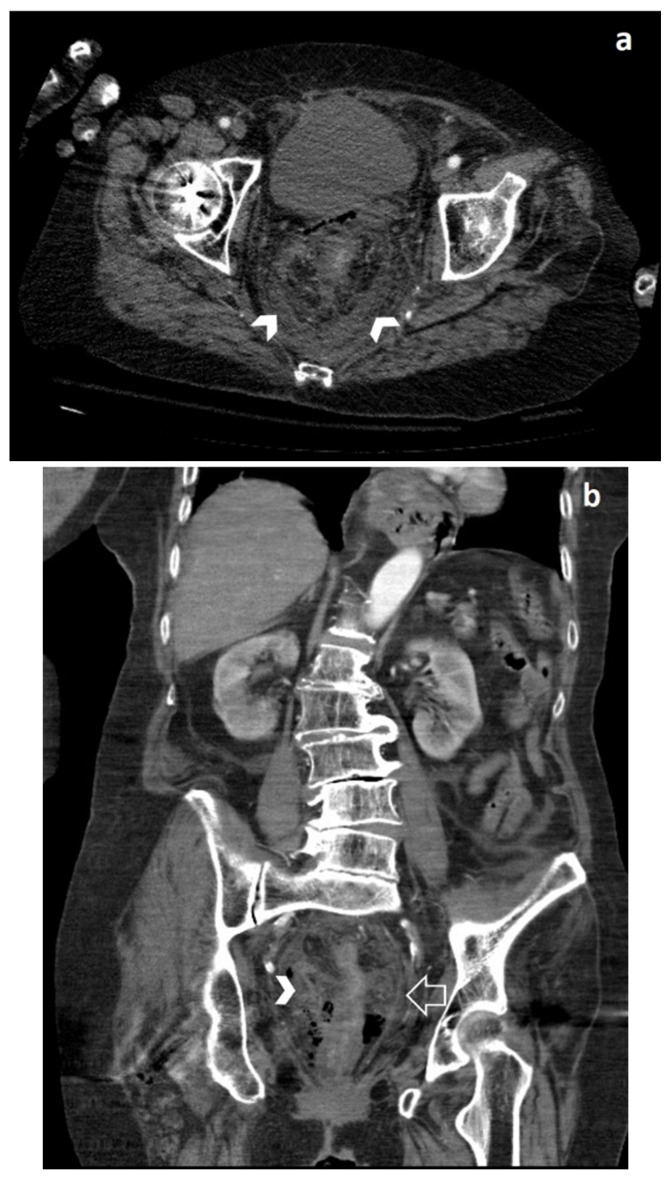
Axial (**a**) and coronal (**b**) CT images show perivisceral fat stranding (arrowheads) and thickening of the mesorectal fascia (open arrow in (**b**)).

**Figure 3:**
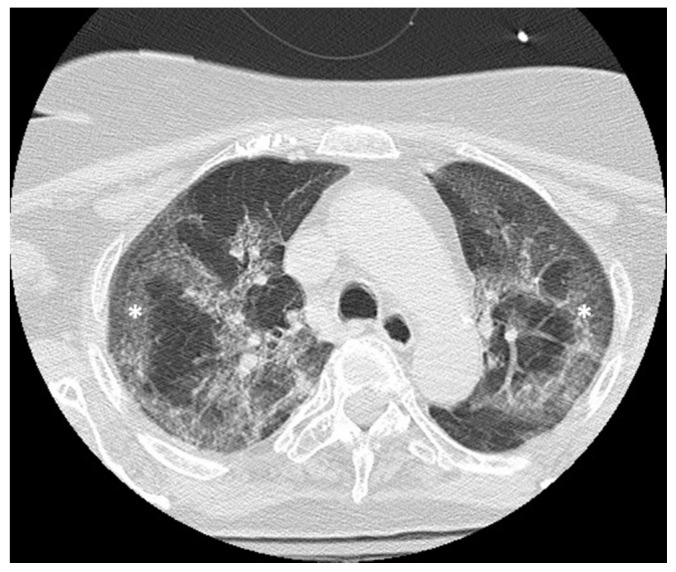
Axial CT image shows ground-glass opacity superimposed by interlobular and intralobular septal thickening (asterisk).

During clinical monitoring, the patient experienced a rapid deterioration of clinical conditions, with the development of septic shock, and so a contrast-enhanced CT of the chest and the abdomen was performed. The abdominal CT showed wall thickening of the lower third of the rectum associated with free perivisceral air (Figure 1), findings suggestive of rectal perforation; perivisceral fat stranding and thickening of the mesorectal fascia were also seen (Figure 2). On the chest CT, typical imaging features of COVID-19 interstitial pneumonia were clearly visible, with patchy bilateral ground-glass opacities associated with interlobular and intralobular septal thickening (Figure 3).

Unfortunately, the patient died 12 h after admission to the hospital.

## 3. Discussion

Large bowel perforation is an abdominal emergency, with an overall mortality of 16.9–19.6% [14]. Etiologies are usually divided into two macrocategories: traumatic vs. spontaneous, which occur due to excessive straining on the anterior rectal wall with a pre-existing condition, such as malignancy, stercoral colitis, ischemic colitis, diverticulitis, inflammatory bowel disease, or infective diseases.

CT is the most valuable imaging technique for identifying the presence, site, and cause of bowel perforation, with an overall accuracy ranging from 82% to 90% [15]. The imaging diagnosis of bowel perforation almost always relies on the detection of free extraluminal air. Although the perforation is often distant from free extraluminal air, the presence of concentrated free air bubbles surrounding a bowel loop is a highly predictive sign for the perforation site. The location of extraluminal air from a rectal perforation depends on its site because the upper two-thirds of the rectum are intraperitoneal, whereas the lower third is extraperitoneal, so free intraperitoneal gas is usually seen if the perforation site is located in the upper two-thirds of the rectum, while if it is located in the lower third, free air may be present in the retroperitoneal space [16]. In addition, focal bowel wall discontinuity, sometimes associated with wall thickening and increased attenuation of the surrounding fat, the so-called “fat stranding”, could be seen. However, the perforation site is visible only in few cases, more often in the upper gastrointestinal tract than in the lower one [15,16].

Lack of bowel wall enhancement, pneumatosis intestinalis, and portomesenteric gas are suggestive of underlying bowel ischemia [15,16,17,18].

Possible causes of COVID-19-related bowel perforation may be related to (1) direct viral infection, given its interaction with the ACE-2 receptors of enterocytes; (2) small vessel thrombosis due to the direct inflammatory effect on vascular endothelium; (3) nonocclusive mesenteric ischemia, which was first hypothesized after values of FC compatible with ischemic bowel disease were detected in COVID-19 patients without gastrointestinal symptoms [8]. According to their anatomy, left flexure and sigmoid colon segments have the highest risk of ischemic colitis, while the distal rectum is usually spared due to its dual blood supply. Of note, bowel wall thickening was detected in 29% of patients with COVID-19, with the colorectal districts being the most affected [11].

In the case herein described, the exact pathophysiological process could not, unfortunately, be determined due to the lack of surgical/bioptical and autopsy samples. However, Bhayana et al. [11] reported a frank discontinuity of a small bowel loop with pneumatosis and a nonenhancing bowel wall on the CT scan, and bowel necrosis with an atypical yellow discoloration of the loops was confirmed by laparotomy. Additionally, in the case reported by Gartland et al. [13], a CT scan showed small bowel ischemia with perforation (but patency of the mesenteric vessels), and, upon surgical examination, a necrotic bowel extending from the ligament of Treitz to the transverse colon, with a perforated terminal ileum. The necrosis, as also described in the case of Bhayana et al., had an atypical bright yellow discoloration rather than black or purple ischemic changes.

On the other hand, in the case reported by De Nardi et al. [12], the CT scan showed free air, distension of the bowel, and the perforation site in the ascending colon. On gross examination, the bowel was enlarged with thin walls. The histological examination showed transmural granulocytic inflammation with fibrinous granulocytic perivisceritis but without vascular thrombosis.

The consistent presence of granulocytes, as reported by the two authors [11,12], may be crucial in understanding the mechanism behind the increase in FC of our patient, given that FC is mainly expressed in neutrophils. Apart from this patient, whose clinical condition deteriorated rapidly, an increase in FC was reported recently in patients without gastrointestinal symptoms or previous history of inflammatory disease [9]. According to these preliminary findings, FC should be assessed in COVID-19 patients on admission, and abdominal imaging might be performed in cases of elevated FC even without gastrointestinal symptoms.

In conclusion, emergency physicians and radiologists should be aware that rectal perforation can be a gastrointestinal manifestation of COVID-19, promptly recognizable by CT.
